# Aberrant anterior tibial artery on knee MRI: A case report advocating for routine search of this anatomical variant

**DOI:** 10.1016/j.radcr.2025.09.053

**Published:** 2025-10-06

**Authors:** Tom Van der Stricht, Philip Van Hover

**Affiliations:** AZORG Hospital, 9300, Aalst, Belgium

**Keywords:** Anterior tibial artery, Aberrant artery, Knee MRI, Popliteus muscle, Vascular variant, Orthopedic surgery

## Abstract

An aberrant anterior tibial artery is an anatomical variant in which the anterior tibial artery arises anomalously from the popliteal artery and takes an atypical anterior course relative to the popliteus muscle. We report a case of a 56-year-old male who underwent a routine knee MRI following trauma to evaluate for a suspected meniscal tear. MRI revealed an incidental finding of an aberrant anterior tibial artery coursing anterior to the popliteus muscle and near the posterior tibial cortex. Knowledge of this variant is crucial to avoid potential iatrogenic vascular injury during orthopedic surgery, especially in procedures involving the proximal posterior tibia, such as high tibial osteotomy, total knee arthroplasty, fracture surgery, lateral meniscal repair, or posterior cruciate ligament reconstruction.

## Introduction

The anterior tibial artery (ATA) normally arises from the popliteal artery. It passes posterior to the popliteus muscle before entering the anterior compartment of the lower leg through the interosseous membrane. However, in a small percentage of the population, the ATA instead arises higher and passes anterior to the popliteus muscle—a configuration termed an aberrant anterior tibial artery (AATA) [1]. Although asymptomatic, this variation is clinically significant during surgical procedures involving the proximal tibia. If unrecognized, the AATA is at considerable risk for injury during surgical interventions such as high tibial osteotomy, total knee arthroplasty, fracture surgery, lateral meniscal repair, or posterior cruciate ligament reconstruction [1,2]. This case illustrates an incidental finding of an AATA on routine knee MRI and emphasizes the importance of always including this anatomical variant in the search pattern.

## Case report

A 56-year-old male with no significant medical history presented after a traumatic injury to the right knee. He complained of medial knee pain. After clinical examination a meniscal tear was suspected, and an MRI of the right knee was performed to evaluate for internal derangement.

MRI was acquired using our routine institutional protocol: axial, coronal, and sagittal proton density fat-saturated (PD FS) sequences, and a sagittal proton density (PD) sequence. No meniscal tear was identified. However, an incidental vascular variant was observed.

**Fig. 1:**
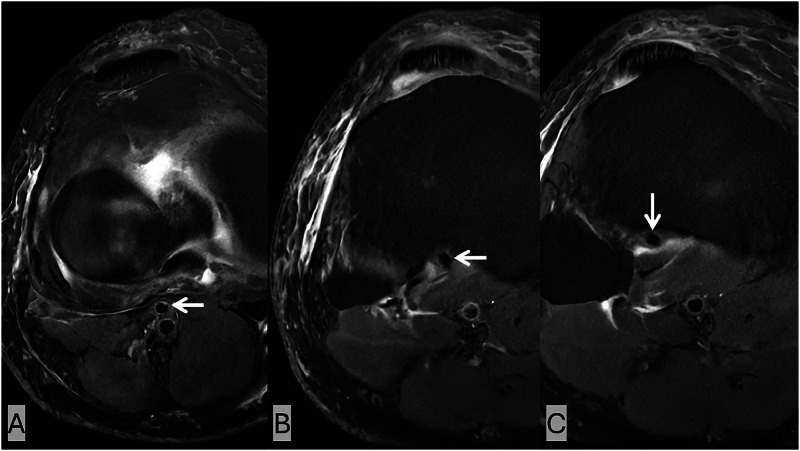
Axial PD FS images at the level of the proximal tibia. (A–C) Sequential axial slices from superior to inferior demonstrate an aberrant anterior tibial artery (white arrows) seen coursing anterior to the popliteus muscle, closely opposed to the posterior tibial cortex without intervening soft tissue. Lateral subcutaneous edema and mild edema in and around the popliteus muscle are also present.

**Fig. 2:**
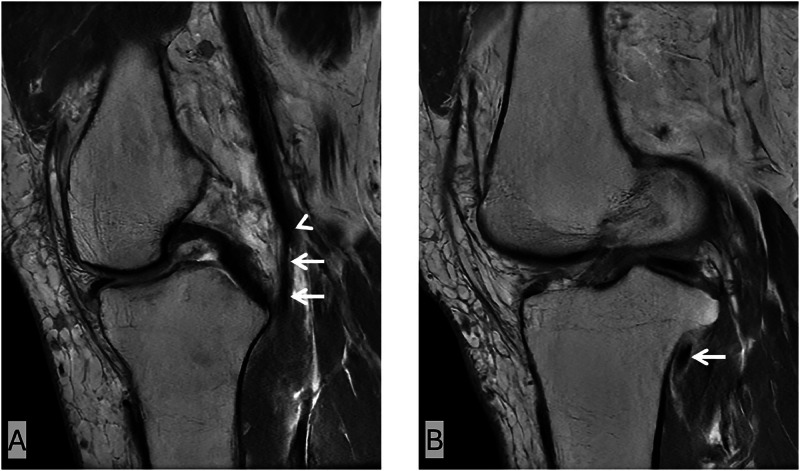
Sagittal PD images of the knee. The aberrant anterior tibial artery (white arrows) has a high origin (white arrowhead) from the popliteal artery and runs in close contact with the posterior cortex of the proximal tibia on sagittal sequences, anterior to the popliteus muscle, and enters the anterior compartment of the leg.

An AATA with a high origin from the popliteal artery was visualized coursing anterior to the popliteus muscle and near the posterior cortex of the proximal tibia, without intervening soft tissue or fat. The vessel was identified as a flow void on multiple sequences, most easily identified on the axial PD FS sequences (Fig. 1). The artery originated from the popliteal artery and traversed anterior to the popliteus muscle before continuing into the anterior compartment. On sagittal PD sequences, this anatomical variant is also well visualized (Fig. 2).

## Discussion

**Fig. 3:**
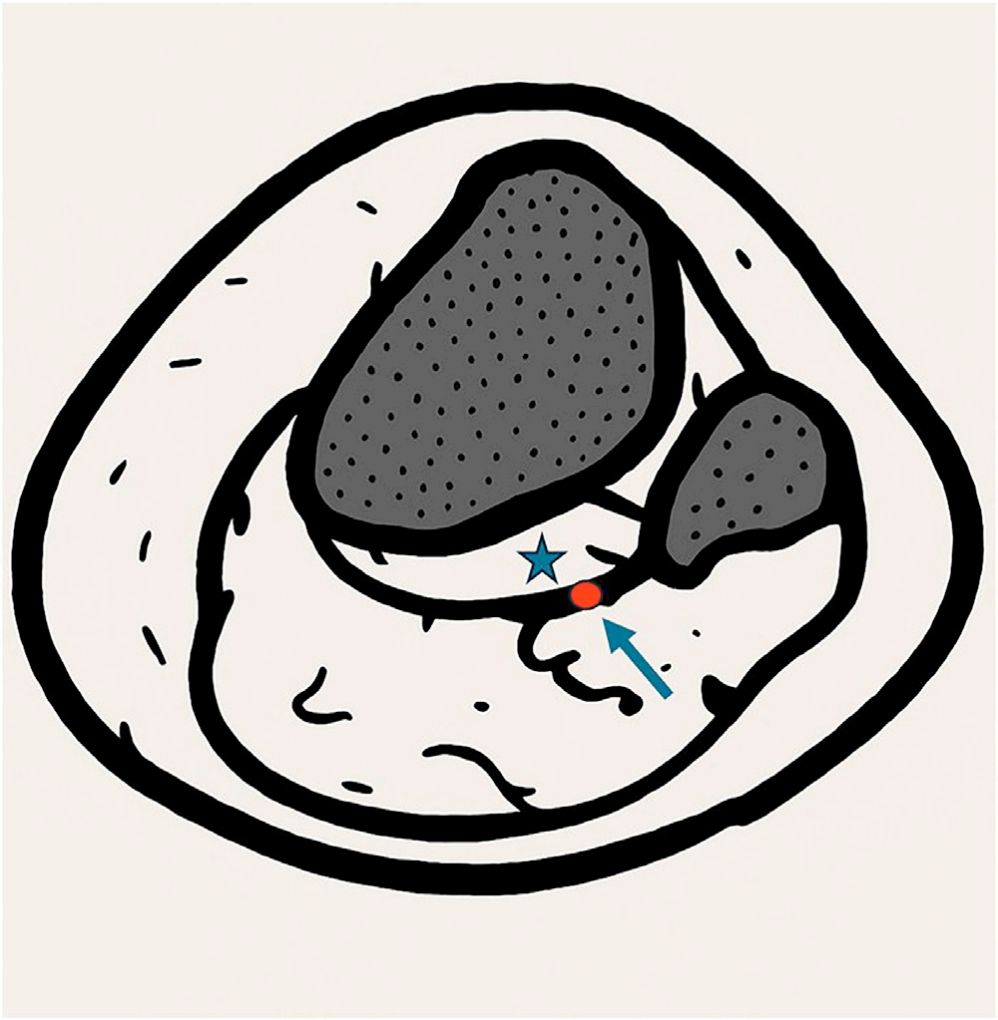
Axial illustration showing the normal anatomy of the popliteal artery (arrow) coursing posterior to the popliteus muscle (star), at the level of the proximal tibia and fibula.

The anterior tibial artery typically arises from the popliteal artery and courses posterior to the popliteus muscle, entering the anterior compartment of the lower leg through the interosseous membrane (Fig. 3). In a small proportion of individuals (2%-3%), the artery instead passes anterior to the popliteus muscle — an anatomical variant referred to as an AATA [1,2]. Most arterial variations in the lower limbs are unilateral; therefore, a normal ATA in one knee does not exclude an AATA in the other knee [1].

The embryological basis of an AATA helps to explain its characteristic course. In early development, the popliteal artery has two components: a superficial segment running posterior to the popliteus muscle and a deep popliteal artery coursing anterior to it. Normally, these vessels are connected by the ramus communicans medius, which develops into the definitive anterior tibial artery, while the deep popliteal artery regresses. If the ramus communicans medius fails to form, the deep popliteal artery persists. In the adult, this persistence appears as a high-origin anterior tibial artery that passes anterior to the popliteus muscle, known as the AATA [1,2].

Though asymptomatic, its clinical relevance lies in its anterior positioning, making it vulnerable during surgical procedures involving the proximal tibia such as high tibial osteotomy, total knee arthroplasty, fracture surgery, lateral meniscal repair or posterior cruciate ligament reconstruction [1,2].

Kim et al. [3] proposed a widely accepted angiographic classification system for popliteal artery branching patterns. In this system, Type I represents the usual popliteal arterial branching pattern, while Type II refers to high bifurcation of the popliteal artery at or above the level of the popliteus muscle. Type II is further divided into subtypes based on the course of the anterior tibial artery. Of these, Type II-A includes cases in which the anterior tibial artery originates high. Within this category, the subtype II-A2 specifically describes a high-origin anterior tibial artery that passes anterior to the popliteus muscle and thus corresponds to the classic AATA. Because Type II-A2 is the only variant in which the anterior tibial artery courses anterior to the popliteus muscle, this type corresponds to the variant observed in our case.

MRI is a non-invasive and highly sensitive imaging modality capable of detecting vascular variants without the need for intravenous contrast [2]. On axial and sagittal PD FS and PD images, the AATA appears as a flow void or vessel structure anterior to the popliteus muscle. Because this anatomical variant is easily identified, radiologists reporting knee MRIs should include it in their “checklist” to avoid potential surgical complications, especially in a preoperative setting.

In addition to MRI, the AATA can also be effectively visualized using Doppler ultrasound. Jordão França et al. [4] reported a case of a patient with intermittent claudication, in whom vascular ultrasound identified a high bifurcation of the popliteal artery with the anterior tibial artery taking an anterior course relative to the popliteus muscle and closely abutting the posterior tibial cortex. This case highlights the potential utility of ultrasound as a non-invasive, accessible, and cost-effective modality for identifying vascular variants such as the AATA, particularly in preoperative settings.

Failure to identify an AATA may result in inadvertent arterial laceration during tibial cuts or drilling, leading to serious vascular complications. These include hematoma formation, pseudoaneurysm, arteriovenous fistula, active hemorrhage, limb ischemia, and the need for emergent vascular repair [1,2]. In some cases, the outcome can be limb-threatening, particularly if intraoperative bleeding is not promptly controlled or the artery is irreversibly damaged. Endovascular interventions, including stenting or embolization, may also be complicated by unrecognized arterial variants.

Therefore, accurate preoperative identification of this vascular variant is essential for surgical planning and patient safety. It should be standard practice to assess for AATA in preoperative imaging studies, particularly in patients undergoing high-risk procedures involving the proximal tibia.

## Conclusion

This case highlights the incidental identification of an AATA on a routine knee MRI following trauma. Recognizing this vascular variant is essential for radiologists and orthopedic surgeons to reduce the risk of intraoperative vascular complications during surgical procedures involving the proximal tibia. Since this anatomical variant is easily seen on MRI, radiologists should always check for it when reporting knee MRIs.

## Patient consent

Written informed consent was obtained from the patient for publication of this case report and accompanying images.
